# Supplementary material for: Scholars180: An effective oral presentation assessment for optometry students
Source: PLoS One. 2023 Jul 24;18(7):e0289081. doi: 10.1371/journal.pone.0289081 (PMC10365299; doi:10.1371/journal.pone.0289081)
Supplement: S1 Appendix — (DOCX) [file pone.0289081.s001.docx]

**S1 Appendix. Rubric for the assignment**

| **Performance Levels/Criteria** | **N (0-29)** | **N (30-49)** | **P (50-59)** | **C (60-69)** | **D (70-79)** | **HD (80-100)** |
| --- | --- | --- | --- | --- | --- | --- |
| **Discipline specific knowledge: theoretical knowledge of ocular anatomy, pathophysiology, signs, and symptoms of eye conditions** | No evidence of anatomical and pathophysiological knowledge of eye conditions. | Little evidence of ocular anatomy and eye conditions.  The content is sometimes relevant but still presents a shallow exploration of the topic. | Acceptable understanding of ocular anatomy, eye diseases, epidemiology, and management options for various eye conditions. | Good understanding of ocular anatomy, eye diseases, epidemiology, and management options various eye conditions. | Very good understanding of ocular anatomy, eye diseases, associated signs and symptoms, epidemiology, and management options of various eye conditions.  The content is relevant and presents a thorough exploration of the topic. | An excellent understanding of ocular anatomy, eye diseases, associated signs and symptoms, epidemiology, and management options of various eye conditions.  The content is all highly relevant and presents a thorough exploration of the topic in both breadth and depth. |
| **Flow** | Does not  introduce the  topic (e.g.  definition/  prevalence).  Did not highlight  the relevance/  importance of  the topic/  problem.  No logical flow.  Does not identify  the details of the eye disease. | Introduces the  topic (e.g.  definition/  prevalence) or  highlights the  relevance/  importance of the eye condition or present the information in  a logical order. | Introduces the  topic (e.g.  definition/  prevalence) to an acceptable degree and  highlights the  relevance/  importance of understanding the eye condition and  identifies crucial  details of the eye disease. | Good introduction of the topic and  attention to details seen through identification of crucial  details of the eye disease. | Very good introduction of the topic and  attention to details seen through identification of crucial  details of the eye disease. | Excellent introduction of the topic and  attention to details seen through identification of crucial  details of the eye disease. |
| **Communication: Structure and presentation** | The presentation is poorly structured that limits the cohesiveness of the material presented. Speaks in low volume and or monotonous volume. | The presentation includes limited information, which limits the cohesiveness of the material presented. | A satisfactory structure that contains required sections. Speaks with satisfactory variation in volume and inflection. | A well-presented and well-structured oral presentation. | A very good presentation that is structured to convey essential points effectively and in a way that is compelling. | An outstanding oral presentation that is clear, consistent, well-presented, and shows an excellent understanding of the eye disease. Holds attention of entire audience.  Speaks with fluctuation in volume and inflection to maintain audience interest and emphasis on key points. |
| **Answering of questions** | Unable to answer questions in a considered and intelligent manner. | Able to answer one of the questions in a considered and intelligent manner. | Able to answer two questions in a considered and intelligent manner. | Able to answer all questions with minimal details. | Able to answer all questions in a considered and intelligent manner. | Able to answer all questions in a methodical and intelligent manner. |
| **Overall 100** | **N**  **0 or above** | **N**  **30 or above** | **P**  **50 or above** | **C**  **60 or above** | **D**  **70 or above** | **HD**  **80 or above** |
