# Supplementary material for: Scholars180: An effective oral presentation assessment for optometry students
Source: PLoS One. 2023 Jul 24;18(7):e0289081. doi: 10.1371/journal.pone.0289081 (PMC10365299; doi:10.1371/journal.pone.0289081)
Supplement: S2 Appendix — (DOCX) [file pone.0289081.s002.docx]

**S2 Appendix. Questions for pre-questionnaire**

**Pre-questionnaire**

**Demographic Information**

1. **What gender do you identify as?**
2. Male
3. Female
4. Other__________
5. Prefer not to answer
6. **How old are you?**
7. 18-24
8. 25-34
9. 35-44
10. Over 45
11. **What is your previous qualification?**
12. Bachelor’s
13. Bachelor’s (Honours)
14. Masters
15. Ph.D. or higher
16. **What best describes your primary status as a student?**
17. On-campus
18. Off-campus
19. Hybrid (combination of traditional classroom experiences and digital course delivery)

**Question 5: Knowledge about eye diseases**

|  | Please rate your level of agreement with each of the statements below | | | | | |
| --- | --- | --- | --- | --- | --- | --- |
| Statements | 1  (very low) | 2  (low) | 3  (medium) | 4  (high) | 5  (very high) | Comments |
| Rate your overall knowledge of most prevalent ocular diseases |  |  |  |  |  |  |
| Rate your knowledge of common treatment methods for various most prevalent eye conditions |  |  |  |  |  |  |
| Rate your diagnostic knowledge of eye conditions. |  |  |  |  |  |  |

**Q6: Attitude and intention towards learning**

|  | Please rate your level of agreement with each of the statements below | | | | | |
| --- | --- | --- | --- | --- | --- | --- |
| Statements | 1  (strongly disagree) | 2  (disagree) | 3 (neutral) | 4 (agree) | 5 (strongly agree) | Comments |
| The current assessment methods in general need improvement to better align with the unit outcomes |  |  |  |  |  |  |
| I am confident about the **signs** of common eye conditions |  |  |  |  |  |  |
| I am confident about the **symptoms** of common eye conditions |  |  |  |  |  |  |
| I am confident about the **treatment** options for common eye conditions |  |  |  |  |  |  |
| I am confident about when to **manage** or refer a patient with an eye condition |  |  |  |  |  |  |

**Q7: Practice**

|  | Please rate your level of agreement with each of the statements below | | | | | |
| --- | --- | --- | --- | --- | --- | --- |
| Statements | 1  (very unlikely) | 2  (unlikely) | 3  (neutral) | 4  (likely) | 5  (very likely) | Comments |
| Imagine you are on placement. As a student Optometrist, a patient with a **chronic eye condition** attends your clinic, how likely are you to appropriately manage the eye disease? |  |  |  |  |  |  |
| Imagine you are on placement. As a student Optometrist, a patient with an **acute eye condition** attends your clinic, how likely are you to appropriately manage the eye disease? |  |  |  |  |  |  |
| Imagine you are on placement. As a student Optometrist, a patient with a **severe eye condition** attends your clinic, how likely are you to appropriately manage the eye disease? |  |  |  |  |  |  |
| Imagine you are on placement. As a student Optometrist, a patient with a **minor eye condition** attends your clinic, how likely are you to appropriately manage the eye disease? |  |  |  |  |  |  |
