# Supplementary material for: Scholars180: An effective oral presentation assessment for optometry students
Source: PLoS One. 2023 Jul 24;18(7):e0289081. doi: 10.1371/journal.pone.0289081 (PMC10365299; doi:10.1371/journal.pone.0289081)
Supplement: S4 Appendix — (DOCX) [file pone.0289081.s004.docx]

**S4 Appendix. Questions for peer evaluation**

**Q8: Assignment organization (peer evaluation)**

|  | 1  (strongly disagree) | 2  (disagree) | 3  (neutral) | 4  (agree) | 5  (strongly agree) |
| --- | --- | --- | --- | --- | --- |
| Most of the presentations were visually appealing |  |  |  |  |  |
| The shared knowledge was relevant to my growth as a student |  |  |  |  |  |
| I understood most of the terminologies used in the oral presentations of my classmates |  |  |  |  |  |
| The content of the presentations met my expectations |  |  |  |  |  |
| I would recommend this assessment to examine future students |  |  |  |  |  |
| Overall, I was satisfied with the **processes** of the assignment |  |  |  |  |  |
| Overall, I was satisfied with the **outcomes** of the assignment |  |  |  |  |  |
